# Probability of Concurrent Deficiency of Vitamin D and Iron in Hypothyroidism: A Cross-Sectional Study

**DOI:** 10.7759/cureus.37152

**Published:** 2023-04-05

**Authors:** Sadia Choudhury Shimmi, Hossameldin f Eldosouky, M Tanveer Hossain Parash, Wan Salman Wan Saudi

**Affiliations:** 1 Department of Biomedical Sciences, Faculty of Medicine and Health Sciences, Universiti Malaysia Sabah, Kota Kinabalu, MYS; 2 Anatomy Unit, Department of Biomedical Sciences, Faculty of Medicine and Health Sciences, Universiti Malaysia Sabah, Kota Kinabalu, MYS

**Keywords:** 25 (oh) vitamin d, uae, iron, vitamin d, bi-deficiency, hypothyroidism

## Abstract

Background: Hypothyroidism is the most common pathophysiological condition that affects mostly females in both developed and developing countries. Data on hypothyroidism among adult females are essential to understand the underactive status of the thyroid gland among the female population and its correlated effects on a deficiency of vitamin D and iron, as effective prevention of osteoporotic changes and iron deficiency anemia is possible. Therefore, the present study was designed to investigate the probability of concurrent iron and vitamin D deficiency among the adult hypothyroid female population of Abu Dhabi, UAE.

Materials and methods: This cross-sectional study was carried out from September 2019 to July 2021 among 500 adult females aged 18 to 45 years old in Sheikh Shakhbout Medical City (SSMC) and Sheikh Khalifa Medical City (SKMC), Abu Dhabi, UAE. After obtaining written informed consent, subjects' demographic characteristics (sun exposure, dressing code, food consumption), anthropometry (height, weight, BMI), and biochemical parameters (thyroid profile, vitamin D profile, iron profile, and blood indices) were measured.

Results: In this study, serum vitamin D and iron levels were significantly (p<0.01) decreased in the hypothyroid female group (study group). The serum vitamin D and iron levels showed a significant negative (p<0.01) correlation with thyroid-stimulating hormone (TSH). Out of 250 study group participants, 61 had a concurrent deficiency of serum vitamin D and iron, yielding a probability (P of low vitamin D and iron and hypothyroidism) of 0.244, which indicates that if 1000 hypothyroid patients are tested for serum vitamin D and iron levels, 24 patients are probable to have low vitamin D and iron.

Conclusion: The study concluded that vitamin D and iron bi-deficiency were observed in adult hypothyroid females in Abu Dhabi, UAE. So, the routine check-up of thyroid function and vitamin D and iron profiles should be done early. Therefore, early vitamin D and iron deficiencies can be detected, and supplements can be given to prevent further health complications like osteoporosis and iron deficiency anemia.

## Introduction

Hypothyroidism is a global endocrine disorder affecting about 5%, undiagnosed about 5%, and more than 99% of individuals have primary hypothyroidism. This hypothyroidism is mainly due to iodine deficiency in the environment; otherwise, with iodine sufficiency, the leading cause is Hashimoto's disease [1]. Among females in the USA and Europe, the prevalence rate of hypothyroidism ranges from 1% to 2%, mainly in older females [2]. However, for United Arab Emirates females, the hypothyroidism prevalence rate has been 6.5% for the last ten years [3]. Furthermore, the hypothyroidism prevalence rate among 20- to 40-year-old females in Libya is 6.18%, and in Saudi Arabia, it is 47.34% [4,5].

Hypothyroidism in females causes several dysfunctions, including goiter, decreased bone density, osteoporosis, anemia, heart diseases, peripheral neuropathy, and mental health problems, and affects reproductive functions with ovarian dysfunction, menstrual variation, delay in pregnancy, and an elevated risk of abortion [6]. In addition, the offspring of hypothyroid females may suffer from congenital abnormalities [7]. Several scientists have reported an association between hypovitaminosis D and hypothyroidism [8-10]. Persian Gulf countries have a high rate of hypovitaminosis D, including Oman (87.5%), Bahrain (86.4%), Qatar (86%), Kuwait (83%), the United Arab Emirates (82.5%), and the Kingdom of Saudi Arabia (81%) [11]. Vitamin D is pivotal for bone homeostasis and mineral balance, controlling calcium, phosphorus, and iron serum levels [12].

Again, Banday et al. [13] and Gökdeniz and Arosio [14] reported an iron deficiency in hypothyroidism patients. Refaat reported that young hypothyroid females in the western province of Saudi Arabia demonstrated iron deficiency in Saudi Arabia [15]. Hypothyroidism causes microcytic anemia due to the malabsorption of iron [16]. Iron in hemoglobin is required for oxygen transport, and myoglobin stores oxygen for muscles and mitochondria for cell respiration [17,18].

Hypothyroidism has been prevalent in the adult female population of Abu Dhabi for the last 13 years. Also, the prevalence of iron and vitamin D deficiency in the United Arab Emirates is significantly high. Therefore, the disease burden will be multiplied if all these diseases are prevalent together, which is most probable given the high prevalence of all these diseases among the female population of Abu Dhabi. Some research on vitamin D deficiency and iron deficiency in hypothyroid patients is available. However, data on the relationship between hypothyroidism, vitamin D, and iron in the UAE are limited to a certain extent. Hence, the present study was designed to investigate the probability of concurrent iron and vitamin D deficiency among the adult hypothyroid female population of Abu Dhabi, UAE.

## Materials and methods

This cross-sectional study was carried out in the Sheikh Shakhbout Medical City (SSMC) and Sheikh Khalifa Medical City (SKMC) hospitals, Abu Dhabi, UAE, from September 2019 to July 2021.

Study population

The population of interest was female UAE citizens 18 to 45 years old from the three districts of Abu Dhabi: Abu Dhabi City, Al Ain, and Al Dhafra. All three district populations have the same lifestyle and food habits.

Inclusion Criteria

Subjects with newly detected hypothyroid cases and thyroid-stimulating hormone (TSH) values > 4.1 µIU/ml were enrolled in the study group, and TSH levels within the normal range were included in the control group by stratified random sampling.

Exclusion Criteria

Patients with post-radioiodine hypothyroidism, liver disorders, renal disorders, primary hyperparathyroidism, autoimmune disorders (Crohn's disease and celiac disease), medications that might alter 25-OH vitamin D and iron metabolism or thyroid functions, vitamin D, iron, calcium, vitamin C, or vitamin B12 supplementation, and menopause were excluded from the study.

Sampling

According to the guidelines of Charan and Biswash [19] for case-control studies for quantitative variables, the sample size was determined. The suggested basis is the following formula:

Sample size = {(r+1)/r}×{SD2(Zβ+Zα/2)2/d2}

= 2×(1.22)2(1.28+1.96)2/(0.31)2

= 124.997 (where r = ratio to control to cases = 1, SD = standard deviation of mean of iron profile in pilot study=1.2, Zβ = standard normal variate for 90% power = 1.28, Zα/2 = standard normal variate for 95% level of significance = 1.96, d = expected mean difference between case and control = 0.50).

The minimum sample size required was 125 subjects in each group. However, the researchers decided to increase the number of enrolled subjects to 250, anticipating a 50% dropout from the study; in total, 500 subjects were selected through a stratified random sampling technique.

Study procedure

According to the research criteria, new suspected hypothyroid subjects based on diagnosis and symptoms (weight gain, sleepiness, fatigue) were enrolled from the general medicine clinic, endocrinology clinic, and laboratory department. The subjects' identities were kept anonymous. Written informed consent was obtained from each participant, and each participant was deidentified by coding. Hence, the authors had n access to information that could identify individual participants during or after data collection.

Each subject filled out a questionnaire with the patient's demographic information, such as date of birth, material status, religion, employment status, addresses, phone numbers, and hospital identity numbers. In addition, symptoms of hypothyroidism included weight gain, decreased appetite, cold intolerance, swollen feet, dry-cold skin, loss or thinning of hair, obstructive sleep apnea, sleepiness, headache, generalized weakness, generalized aches and pains, muscle weakness, muscle pain, bone pain, change of mood, constipation, and lethargy.

The questionnaire also included data on family history, social history, food consumption (a regular/fatty diet), sun exposure, standard dress code, physical activities, and supplementation. Furthermore, the questionnaire included a checklist of physical examinations focusing on blood pressure, pulse rate, pallor, edema, height/length, weight, neck swelling, and a systematic review. The time needed to complete the questionnaire was 15 minutes.

Anthropometry

An electronic patient weighing scale measured the weight and height of young females without shoes and with regular clothes (Seca model 285). The sensor recorded the height of the participant in a straight position.

The body mass index was calculated using BMI = [weight/height^2^ (m^2^)]. The Centers for Disease Control and Prevention (CDC) considers a BMI below 18.5 to be underweight. A BMI of 18.5 to 24.9 is considered normal, 25 to 29.9 overweight, and 30 and above obese.

Collection of blood and biochemical assessment

A 5 ml venous blood sample was collected in light green Li-heparin tubes (for plasma) with a unique barcode for every patient. The specimen was sent to the laboratory receiving area instantly in a plastic biohazard specimen bag, firmly closed, and accompanied by the request form in an external sheath. A Hettich Rotina 380 centrifuge centrifuged the sample for 10 minutes. Then, the sample was sent to the clinical chemistry section in the specimen rack, immediately processed, and kept in a sorted retrieval sample rack for one week in the refrigerator (ALS Refrigerator FRL 500 V-GL-M Lab, Single Door).

The researchers performed the biochemical analysis of TSH, free thyroxine (FT4), free triiodothyronine (FT3), thyroid peroxidase (TPO) Abs, Vit D, iron, transferrin, transferrin saturation, total iron binding capacity (TIBC), and ferritin on a ROCHE COBAS 8000 chemistry analyzer. In addition, hemoglobin (Hb), packed cell volume (PCV), mean corpuscular volume (MCV), mean corpuscular hemoglobin (MCH), and mean corpuscular hemoglobin concentration (MCHC) were analyzed with the Sysmex XN-3100.

Statistical analysis

**Figure 1:**
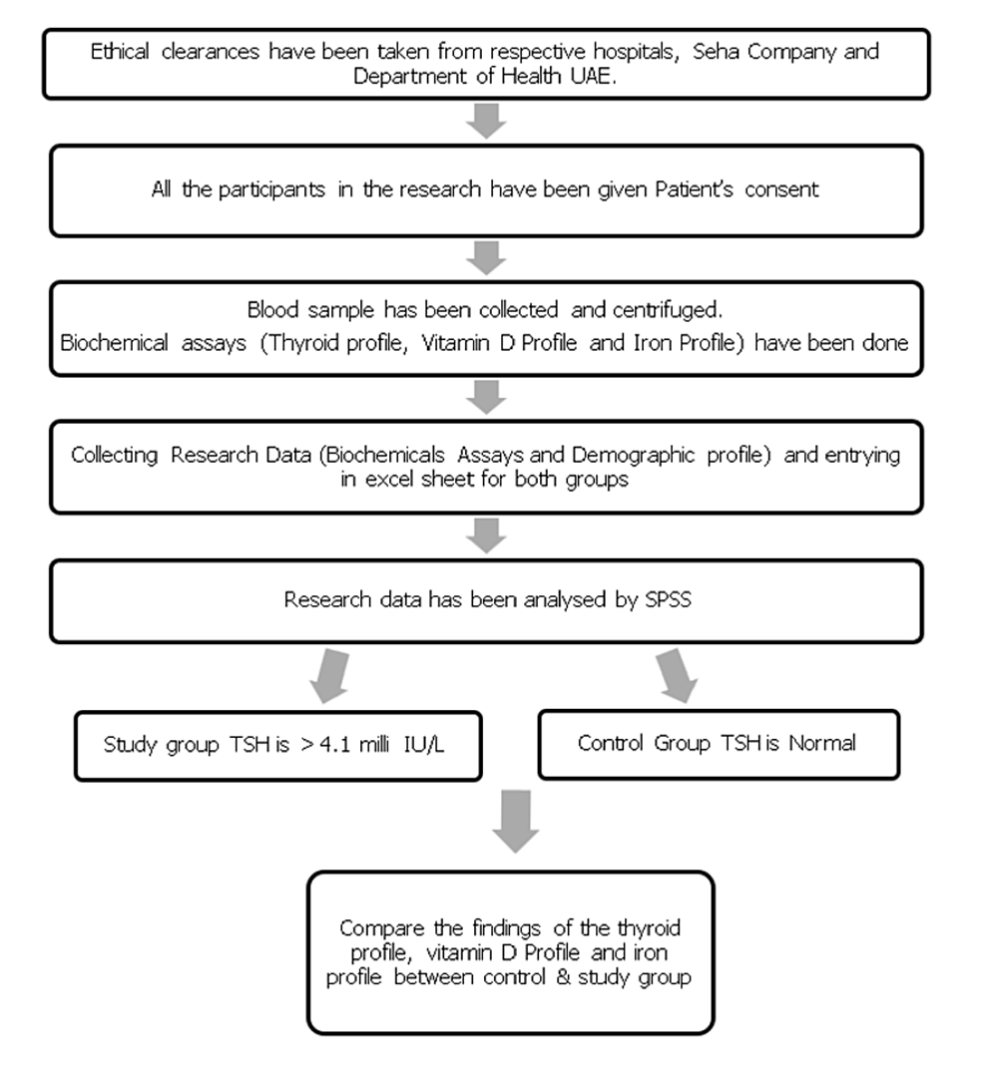
Flow chart

Statistical Package for Social Science (SPSS) version 27.0 (IBM Corp., Armonk, NY) was used to analyze the data. Descriptive statistics were used to describe the participants' locations, age distribution, BMI, sun exposure, dress code, and nutritional status. Furthermore, unpaired t-tests were used to evaluate the difference in means of thyroid profiles (TSH, free T3, free T4, and anti-TPO antibodies), vitamin D levels, iron profiles (serum iron, transferrin, TIBC, transferrin saturation, and ferritin), and blood indices (Hb, PCV, MCV, MCH, and MCHC) among the control and study groups. Data are expressed as the mean or difference of the mean with a 95% confidence interval (CI) to infer the findings in the population. In addition, Pearson correlation tests were used to test the correlation between iron and vitamin D with the thyroid profile and between the thyroid profile, BMI, vitamin D, and iron with food status. Figure 1 demonstrates the flow of the study procedure.

## Results

Distribution of age and BMI of the participants

The age of the participants ranged from 18 to 45 years. The mean age was 31.2. The lowest BMI was 15.2, and the highest was 58.9, with a mean of 29.2 (Table 1).

**Table 1:** Distribution of age and BMI among the participants (n=500)

	Minimum	Maximum	Mean	Std. deviation
Age (years)	18	45	31.2	7.89
BMI (kg/m^2^)	15.2	58.9	29.2	6.70

**Figure 2:**
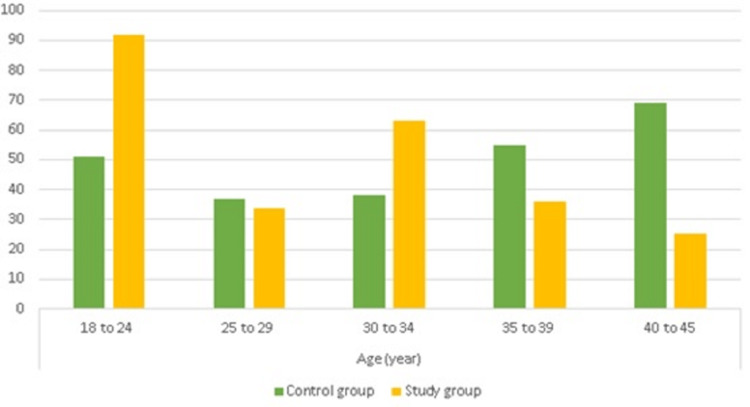
Age distribution among the control and study groups (n=500)

Figure 2 shows that the highest percentage of participants in the study group was between 18 and 24 years old, and in the control group, it was between 40 and 45 years old. The lowest percentage of participants in the study group was between 40 and 45 years old, and the control group was 25 to 29 years old.

**Figure 3:**
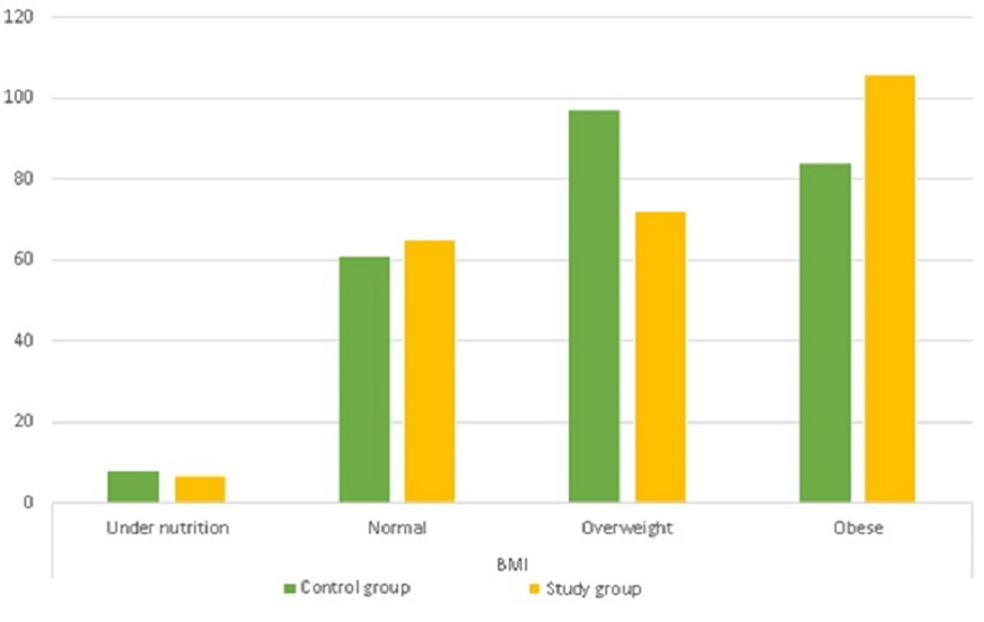
BMI distribution among the control and study groups (n=500)

In terms of BMI distribution, the majority of the participants in the study group were obese (42%), followed by overweight (30%), normal (26%), and underweight (2%). In the control group, most of the participants were overweight (38%), followed by obese (34%), normal (24%), and underweight (4%) (Figure 3).

**Figure 4:**
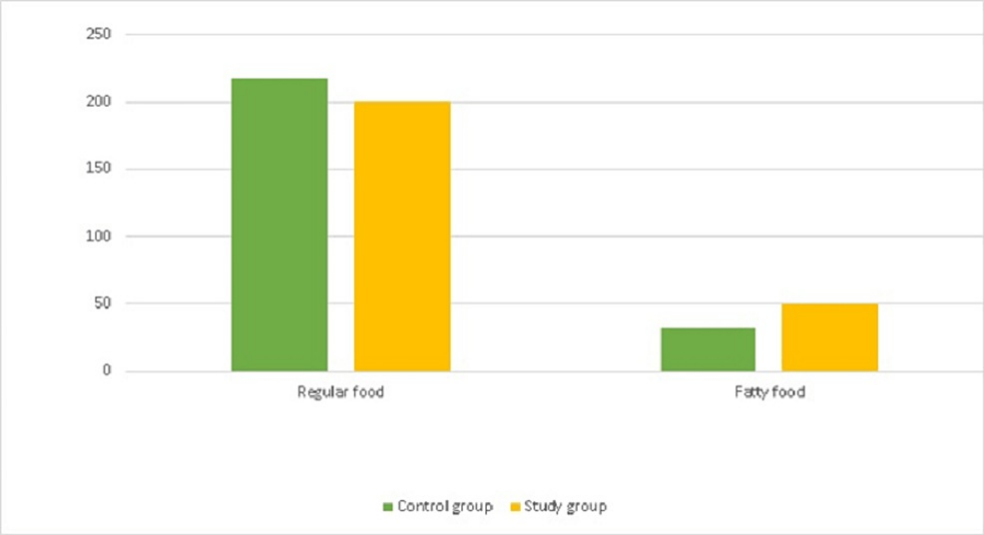
Food consumption among the control group and study group (n=500)

Figure 4 shows that both the control and study groups showed highly regular food consumption (home diet) and low consumption of fatty foods.

Distribution of symptoms among study group participants

Out of 250 study group participants, 202 subjects experienced generalized weakness, 152 gained weight, 84 had musculoskeletal pain, 44 had headaches, and 26 showed intolerance to colds (Table 2).

**Table 2:** Distribution of symptoms among the study group in different age groups (n=250)

Age (grouping)	Weight gain	Generalized weakness	Musculoskeletal pain	Cold intolerance	Headache
18–24 years	29	69	24	9	12
25–29 years	16	35	16	3	5
30–34 years	23	50	17	6	13
35–39 years	17	28	15	4	10
40–45 years	13	20	12	4	4
Total	152	202	84	26	44

Differences in thyroid profile between the study group and the control group

The TSH level was −10.1, with a 95% confidence interval ranging from −12.65 to −7.57. Free T4 was 2.9, whereas the 95% confidence interval lower bound was 2.41 and the upper bound was 3.41. Furthermore, the difference in free T3 was 0.2 (95% CI: lower bound 0.07, upper bound 0.33). The difference in thyroid peroxidase antibodies was −211.9, with a 95% confidence interval ranging from −241.66 to −1.82.18 (Table 3).

**Table 3:** The difference in thyroid profile between the study and control groups (n=500) *Significant at p<0.01 level

Thyroid profile (Unit)	Mean (±SD)		Mean difference	t	df	p-value	95% CI	
	Control group	Study group					Lower	Upper
TSH (milli IU/L)	2.06 (±1.04)	12.2 (±20.42)	−10.1	−7.817	498	0.000*	−12.65	−7.57
FT4 (pmol/L)	15.6 (±2.39)	12.7 (±03.23)	2.9	11.477	498	0.000*	2.41	3.41
FT3 (pmol/L)	4.6(±0.62)	4.4 (±00.81)	0.2	3.086	498	0.002*	0.07	0.33
TPO-Abs (IU/ml)	9.6 (±1.98)	221.5(±239.35)	−211.9	−13.999	498	0.000*	−241.66	−182.18

Differences in vitamin D and iron profile among the study and control groups

Table 4 shows that the difference in vitamin D level was 38.70, with a 95% confidence interval ranging from 36.33 to 41.16. The difference in serum iron level was 8.8, with a 95% confidence interval ranging from 8.16 to 9.40. The mean difference in serum TIBC levels was −0.5 (95% CI: lower bound −0.58, upper bound −0.45). The difference in transferrin level was −12.1, whereas the 95% confidence interval lower bound was −13.66 and the upper bound was −10.48. Moreover, the mean difference in transferrin saturation % was 0.2, with a 95% confidence interval ranging from 0.14 to 0.17. The difference in serum ferritin level was 35.3 (95% CI: lower bound 31.73, upper bound 38.79).

**Table 4:** Serum vitamin D and iron profile among the study and control groups (n=500) *Significant at p<0.01 level

Vitamin D and iron profile (Unit)	Mean (±SD)		Mean difference	t	df	p-value	95% CI	
	Control group (250)	Study group (250)					Lower	Upper
Vit D (nmol/L)	67.9 (±16.69)	29.2 (±10.73)	38.70	30.834	498	0.000^*^	36.23	41.16
Iron (μmol/L)	13.1 (±4.84)	4.4 (±1.22)	8.8	27.812	498	0.000^*^	8.16	9.40
TIBC (μmol/L)	2.6 (±0.27)	3.2 (±0.47)	−0.5	−14.993	498	0.000^*^	−0.58	−0.45
Transferrin (g/L)	61.9 (±6.41)	74.0 (±11.10)	−12.1	−14.889	498	0.000^*^	−13.66	−10.48
Transferrin saturation (%)	0.2 (±0.08)	0.06 (±0.02)	0.2	28.577	498	0.000^*^	0.14	0.17
Ferritin (μg/L)	43.7 (±28.22)	8.4 (±3.32)	35.3	19.622	498	0.000^*^	31.73	38.7

Correlation of iron and vitamin D with thyroid profile

Serum iron and vitamin D levels negatively correlate with TSH (Table 5). The probability of a hypothyroid person having both vitamin D and iron deficiency has been demonstrated in Table 6.

**Table 5:** Correlation of serum iron and vitamin D with TSH (n=500) *Correlation is significant p<0.01 level

	Correlation coefficient (with TSH)	p-value
Serum iron	−0.317	0.000^*^
Serum vitamin D	−0.276	0.000^*^

**Table 6:** Probability of serum low vitamin D, iron, and both among the study group (n=250)

Serum level of		Study group	Probability
Vitamin D	Normal	163	-
	Low	87	0.348
Iron	Normal	160	-
	Low	90	0.360
Both vitamin D and iron	Normal	134	-
	Low	61	0.244

## Discussion

Age and BMI

In the present study, the highest percentage of participants for the study group were between 18 and 24 years old. This finding was consistent with other studies of Arab countries [20-23]. In addition, researchers from other countries agreed with this finding [10,24-27].

In contrast, Refaat [15] in Saudi Arabia revealed that there was an age difference (p=0.01) between the control and study groups. Kim [28] conducted a study in the Republic of Korea and observed the difference between the two groups. This finding may be due to different populations and countries. In this study, most participants were obese (42%), and in the control group, most participants were overweight (38%). In this study, more overweight and obese people were observed in the control group due to their lifestyle and food habits and fewer physical activities. Various researchers from Arab countries corroborated the finding of obesity in the study group of females [3,29]. Likewise, researchers from other countries observed similar findings [30,31]. Moreover, some studies showed normal BMI in the study group. For example, Aboud et al. [21] in Iraq, Kim [28] in the Republic of Korea, and ElRawi et al. [32] in Egypt observed a normal BMI in the study group. This discrepancy may be due to the lifestyle, food habits, and physical activities of people in different countries.

However, overweight or obese individuals were found in the control group in some studies, which supports our results. For example, in the United Arab Emirates, Belal [33] reported obesity in healthy individuals (the control group). In Greece and Egypt, Mazokopakis et al. [34] and ElRawi et al. [32], respectively, reported overweight subjects in the control group. This finding may be due to the participants' food habits, lifestyle, and reduced physical activities.

Food consumption

This study showed high consumption of regular food (meat machbous, chicken machbous, meat harese, chicken harese, meat salona, maleh, ragag, gurus, legemat, and balaleet) for both the study and control groups. These findings are consistent with those of other investigators [35].

Thyroid profile

Our study showed elevated mean TSH levels in the study group, and the levels were significantly (p<0.01) higher in the study group than in the control group. Some researchers supported these findings, reporting TSH levels of 12 milli IU/L [26,27]. Other researchers reported a TSH level of <8 milli IU/L in the study group [20,22]. This means that the TSH level increased but was not relatively high. The TSH result varies because of different inclusion criteria for the study subjects. The participants in those studies were previously diagnosed as hypothyroid patients and came for follow-ups or routine check-ups for thyroid function. Moreover, Dahiya et al. [24], ElRawi et al. [32], and Fawzy et al. [36] observed that the TSH level was >25 milli IU/L in the study group. This observation was due to the study group comprising autoimmune hypothyroid patients with hypothyroidism symptoms or untreated with L-thyroxine. The present study showed elevated mean TPO-Ab levels in the study group, which were significantly (p<0.01) higher than in the control group. Other studies have supported this finding [24,26,28]. Therefore, this elevation of TPO-Abs may be because the females in the study group were autoimmune hypothyroid patients. An average mean FT4 level was observed in the study group. Moreover, FT4 was significantly (p<0.01) lower in the study group than in the control group. This typical result of FT4 could be explained because the study populations were newly detected or came for routine check-ups of thyroid function. Researchers from other countries have shown similar results [24,26,28].

Again, Fawzy et al. [36] reported that FT4 was 6.56 pmol/L in the study group. This below-average level of FT4 in the study group was due to the subjects being either autoimmune hypothyroid patients with hypothyroidism symptoms or untreated with L-thyroxine. In the present study, the mean FT3 level in the study group was significantly (p<0.01) lower than in the control group. Other researchers agreed with this finding [10,24,26]. Furthermore, there were nonsignificant TSH differences and free T4 and thyroid peroxidase antibodies among the different age groups in the study group. However, there was a significant difference in free T3 (p<0.01) among the age groups in the study group.

Vitamin D

The present study showed decreased mean vitamin D levels in the study group, which were significantly (p<0.01) lower than those in the control group. This finding has been supported by different researchers from different countries [22,23,27,28,34,37,38]. Furthermore, there were no significant differences in vitamin D between the different age groups in the study group.

Iron profile

In this study, there were significantly (p<0.01) decreased mean iron levels in the study group compared with the control group. This finding was consistent with the findings of some researchers [13,24,39,40]. In addition, the mean serum TIBC level and transferrin level were significantly (p<0.01) higher in the study group than in the control group in the present study. However, transferrin saturation % and serum ferritin were significantly (p<0.01) lower in the study group than in the control group. These findings were supported by Dahiya et al. [24]. Furthermore, there were no significant differences in the iron profile between the different age groups in the study group.

Moreover, our study found significant (p<0.01) decreases in Hb, PCV, MCV, MCH, and MCHC in the study group. Due to the lower iron status observed in the study group, other blood index parameters decreased as well. These parameters suggest iron deficiency anemia in the hypothyroid female group, which was not confirmed in this study. These findings were supported by Dorgalaleh et al. [16] and Maheshwari et al. [41].

Correlation between the TSH, vitamin D, and iron

Serum vitamin D has a significantly (p<0.01) high negative correlation with TSH. Furthermore, serum vitamin D decreased when the TSH level increased and vice versa. Various researchers from different countries have observed a similar correlation between vitamin D and TSH [25,27,37]. These results suggested that there may be a significant association between vitamin D deficiency and hypothyroidism. Furthermore, Patni et al. [26] found no significant correlation between vitamin D and TSH. This discrepancy was due to the study group subjects having Hashimoto's thyroiditis and Graves' diseases.

Moreover, serum iron had a significantly (p<0.01) high negative correlation with TSH. Serum iron levels decreased when TSH levels increased, and vice versa. Dahiya et al. [24] and Bandey et al. [13] found a similar finding. These results suggested that there may be a significant association between iron deficiency and hypothyroidism.

Bideficiency of vitamin D and iron

None of the participants in the control group had serum vitamin D or iron deficiency. However, out of 250 study group participants, 61 had a concurrent deficiency of serum vitamin D and iron, yielding a probability (P of low vitamin D and iron and hypothyroidism) of 0.244, which indicates that if 1000 hypothyroid patients are tested for serum vitamin D and iron levels, 24 patients are probable to have low vitamin D and iron. In contrast, as per Table 6, in 1000 hypothyroid patients, 35 study group subjects had the probability of developing hypovitaminosis D alone, and 36 had the probability of developing low serum iron levels. Hence, we can draw inferences from the findings of Table 6 that hypothyroidism could be associated with deficiencies of vitamin D and iron. However, this finding could not be compared with other studies due to insufficient data.

Possible mechanism of hypothyroidism with serum vitamin D

In hypothyroid patients, vitamin D levels decrease due to inactivation and malabsorption from the intestine. Both vitamin D and thyroid hormone bind to similar steroid hormone receptors. Polymorphism of the vitamin D receptor causes autoimmune thyroid diseases. Moreover, Hashimoto's thyroiditis inactivates thyroid peroxidase and is recognized as an antigen, leading to intrathyroidal lymphocytic infiltration and the formation of TPO-Abs. Hashimoto's thyroiditis also leads to proinflammatory cytokine formation (increased Th1/Th2 ratio) [42]. Again, vitamin D deficiency decreases the immunomodulatory effect on T cells [43].

Moreover, an increased Th1 ratio that enhances cell apoptosis in Hashimoto's thyroiditis also increases Th1 and elevates B cell antibody production to enhance anti-apoptotic molecules, leading to the death of cytotoxic lymphocytes and thyroid tissue infiltration [43].

Possible mechanism of hypothyroidism with serum iron

In hypothyroid patients, the iron level decreases due to the under-activation of erythropoietin (EPO) gene expression, affecting EPO secretion [44]. Iron deficiencies decrease the activity of thyroid peroxidase (heme-dependent) [14]. Moreover, hypothyroidism leads to gut malabsorption of iron due to reduced digestive acids [24]. Our study showed a deficiency of vitamin D and iron in adult hypothyroid females. The newly diagnosed hypothyroid female had a significant decrease in vitamin D and iron levels. This study found that in the UAE, females at a significantly younger age (18 to 24 years) developed hypothyroidism and eventually both vitamin D and iron deficiency. Therefore, these female populations experienced vitamin D and iron-related complications from early adulthood.

Due to vitamin D deficiency, there may be osteoporotic changes in hypothyroid female subjects. However, these changes were not evaluated. The iron profile was measured, but peripheral blood film for iron deficiency anemia could not be detected in the study group. Therefore, this study was limited to investigating the association between hypothyroidism and vitamin D and iron deficiencies. Future studies should focus on determining the pathophysiology of the association.

Limitations

The selection of the subjects of this research was limited to three districts of Abu Dhabi. The osteoporotic changes due to vitamin D deficiency in the hypothyroid female subjects, the peripheral blood film for iron deficiency anaemia in the anaemic patients, and the underlying causes of obesity in the morbid patients were not evaluated. Furthermore, this study was cross-sectional, so it was limited to detecting the association between hypothyroidism and vitamin D and iron but not the causation.

In future work, osteoporotic changes and iron deficiency anaemia can be evaluated in the hypothyroid population. Further study can be performed by adopting an experimental design to explore the causation. Future studies should further explore whether hypothyroidism causes vitamin D and iron deficiencies or vice versa.

## Conclusions

Our study investigated adult hypothyroid females (18 to 45 years) vitamin D and iron status in Abu Dhabi, UAE. It can be concluded that a bi-deficiency of vitamin D and iron was observed in the adult hypothyroid female population in Abu Dhabi, UAE. So, vitamin D and iron should be included in routine check-ups for thyroid function tests. This study also found that 18 to 25 years of females were the highest in the hypothyroid group. So, the routine check-up of thyroid function, vitamin D, and iron profile should be done from the early age of 18. Therefore, early vitamin D and iron deficiencies can be detected, and supplements can be given to prevent further health complications like osteoporosis and iron deficiency anaemia.
